# Ginseng as a Functional Food Intervention for Interstitial Lung Disease: A Review of the Benefits and Molecular Mechanisms

**DOI:** 10.1002/fsn3.72265

**Published:** 2026-08-16

**Authors:** Jiahui Li, Guixin Di, Haijiao Wang, Mengzhu Chen, Keju Wang, Ziyuan Wang, Yanxin Li, Tan Wang

**Affiliations:** ^1^ Changchun University of Chinese Medicine Changchun China; ^2^ Affiliated Hospital of Changchun University of Chinese Medicine Changchun China; ^3^ Medical School Tianjin University Tianjin China

**Keywords:** dietary supplementation, functional foods, ginseng, interstitial lung disease, signaling pathway

## Abstract

Interstitial lung disease (ILD) encompasses a complex group of disorders primarily characterized by inflammation and fibrosis of the pulmonary interstitium. Affected individuals often present with dyspnea and cough, and their quality of life is markedly reduced. Given the limitations of current health management strategies, there is growing interest in exploring adjunctive supportive approaches based on functional foods. Ginseng, a traditional medicinal and edible plant, has a long history of consumption in East Asia. Studies have shown that ginseng and its major bioactive constituents, such as ginsenosides, exert beneficial regulatory effects on lung health by modulating key signaling pathways, including TGF‐β/Smad, NF‐κB, Nrf2/HO‐1, and MAPK. These effects primarily involve health‐promoting properties such as anti‐inflammatory, antioxidant, and immunomodulatory activities. From the perspective of food science and nutrition, this article systematically reviews the regulatory mechanisms through which ginseng and its active components influence pathological processes related to ILD. It also discusses its traditional use as a functional food, dietary exposure, safety, and intake considerations. Current evidence from preclinical studies and early‐stage population‐based investigations supports the potential of ginseng as a supportive dietary component for maintaining lung health. Future research should include well‐designed nutritional studies to clarify its specific role in health management and to establish appropriate intake regimens.

## Graphical abstract

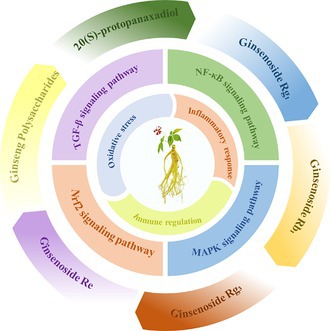

## 1 Introduction

Interstitial Lung Disease (ILD) is a group of chronic pulmonary disorders characterized by inflammation and fibrosis of the pulmonary interstitium. There are estimated to be over 200 distinct subtypes, including Idiopathic Pulmonary Fibrosis (IPF) and Connective Tissue Disease‐associated ILD (CTD‐ILD). IPF is estimated to account for approximately one‐third of all ILD cases, connective tissue disorders for 25%, and allergic pneumonias for 15% (Maher 2024). The incidence of ILD is rising annually, affecting approximately 650,000 individuals in the United States and resulting in approximately 25,000 to 30,000 deaths each year (Jeganathan and Sathananthan 2022). Furthermore, ILD represents the most common cause of mortality in patients with systemic sclerosis, with a 10‐year mortality rate reaching 40% (Perelas et al. 2020). Patients diagnosed with ILD experience significantly reduced quality of life, often presenting with symptoms such as dyspnea and cough (Jeganathan and Sathananthan 2020). Untreated individuals typically have a median survival of a mere 3–5 years (Wijsenbeek et al. 2022). Pirfenidone and nintedanib, the current mainstay treatments, can only slow disease progression without providing a cure; moreover, they are costly and associated with significant adverse effects (Flaherty et al. 2019; King et al. 2014). Given the high mortality of ILD and the limited efficacy and accessibility of current management options, there is an urgent need to identify safe and readily accessible health interventions as early as possible.

In recent years, natural products such as ginseng have attracted considerable attention due to their beneficial health effects and favorable safety profile and have gradually become popular dietary supplements worldwide. Ginseng is a perennial herbaceous plant of the Araliaceae family. It is native to East Asian regions, including China and Korea, and is predominantly distributed in the temperate zones of East Asia (Coon and Ernst 2002). Ginseng is a plant with dual food and medicinal uses, possessing immune‐enhancing properties. As a health‐promoting food, it is increasingly incorporated into everyday dishes, beverages, teas, sweets, and other food products (Kim, Kim, Jo, et al. 2022). The medicinal value of ginseng is attributed to the various parts of the plant, including the leaves, flowers, fruits, roots, rhizomes, and fibrous roots. The dried roots and rhizomes are primarily employed in traditional medicine, with documented clinical applications dating back approximately 5000 years (Mancuso and Santangelo 2017). Ginseng is characterized by a high concentration of active components, including ginsenosides and polysaccharides, which have been demonstrated to possess a wide range of biological activities. These include anti‐inflammatory, antioxidant, immunomodulatory, anti‐fibrotic, and tissue repair‐stimulating activities (Lee et al. 2025). As a promising functional food ingredient, ginseng demonstrates considerable potential for improving health outcomes associated with ILD. Research indicates that the ginsenoside metabolite 20(S)‐protopanaxadiol (PDD) directly alleviates PF by modulating the Adenosine 5′‐monophosphate‐activated protein kinase (AMPK)/stimulator of interferon genes (STING) pathway and reprogramming glycolytic metabolism. Furthermore, ginseng may exert an indirect influence on PF by modulating gut microbiota and their metabolites, thereby regulating targets such as Glucose‐6‐phosphate dehydrogenase (G6PD) and Sphingosine kinase 1 (SPHK1) (Ruan et al. 2024). Various ginseng preparations can alleviate fatigue and significantly improve patients' quality of life, thereby exerting beneficial health‐promoting effects (Li, Hou, et al. 2022).

**FIGURE 1:**
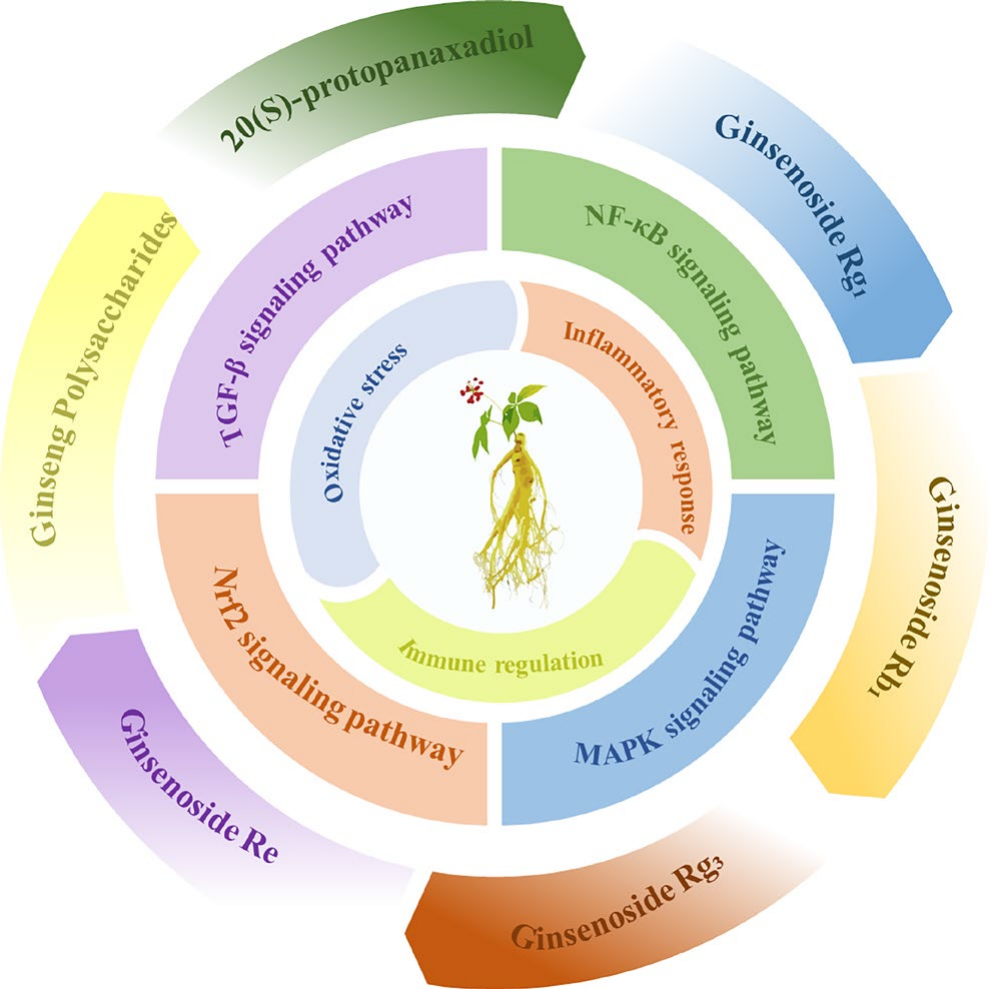
Overview of the regulatory effects of Ginseng and its active components on ILD‐related pathological processes.

From the perspectives of food science and nutrition, this article reviews the regulatory mechanisms through which ginseng and its major components influence ILD‐related pathological processes and discusses its potential as a functional food and future research directions (Figure 1).

## 2 Overview of ILD

### 2.1 The Physiological Function of the Pulmonary Interstitium

ILD development involves multiple interconnected pathways, including inflammation, oxidative stress, and immune regulation (Koudstaal et al. 2023). The pulmonary interstitium performs several vital functions, including maintaining the structural integrity of the alveoli, facilitating gas exchange, and regulating immune processes. The disruption of these functions constitutes a fundamental basis for the onset and progression of ILD (Philp et al. 2018; Parker et al. 2014). The pulmonary interstitium, as the connective tissue linking alveoli and blood vessels, is primarily composed of fibroblasts, collagen, elastic fibers, and matrix proteins. It has been demonstrated that the structure provides a foundation for the lungs and ensures that alveoli maintain their normal morphology during respiratory movements (Klein 2021). In particular, the function of fibroblasts is to participate in the production and remodeling of the extracellular matrix (ECM). Collagen and elastic fibers are the principal structural elements that endow lung tissue with its elasticity and compliance, thus facilitating the recurrent cycles of expansion and contraction that are integral to the respiratory therapeutic direction process (Duhig 2022; Panagiotidis et al. 2025). In addition to their structural functions, pulmonary interstitial cells modulate the immune microenvironment by means of the secretion of cytokines. They participate in local immune surveillance and inflammatory responses. This contributes to the protection of lung tissue from external pathogens and harmful substances (Wu et al. 2025; Ma 2020).

The hallmark pathological features of PF are abnormal activation of pulmonary interstitial cells and excessive deposition of the ECM. This finding suggests that the pulmonary interstitium fulfills a dual role in lung diseases, providing structural support and contributing to the pathogenesis and progression of these diseases (Confalonieri et al. 2022). Recent studies have indicated that Tripartite motif‐containing protein 72 exerts an anti‐fibrotic effect by indirectly influencing fibroblast activation and ECM deposition by preserving epithelial cell integrity. This action thereby reduces inflammatory cell infiltration and tissue damage (Cong et al. 2020). This further underscores the critical influence of the pulmonary interstitial microenvironment on lung tissue repair and regeneration, with maintaining its homeostasis being pivotal to preserving pulmonary health. Bioactive compounds in foods, such as the saponins and polysaccharides found in ginseng, can provide nutritional support for maintaining pulmonary interstitial homeostasis by regulating the function of interstitial cells and promoting a balanced ECM microenvironment.

### 2.2 Pathogenesis of ILD

The onset and progression of ILD involve multiple complex mechanisms, including inflammation, oxidative stress, and immune dysregulation. These pathological processes represent the key biological basis for the health‐promoting effects of dietary components such as ginsenosides.

#### 2.2.1 The Role of Inflammatory Responses in ILD

In the pathological progression of ILD, the infiltration of inflammatory cells such as macrophages and lymphocytes is a persistent feature. The release of substantial inflammatory mediators by these cells collectively drives the development of the disease (Kishore and Petrek 2021). Research indicates that NOD‐like receptor family pyrin domain‐containing 3 (NLRP3) inflammasomes are activated within the ILD microenvironment. The maturation and release of cytokines, such as Interleukin‐1β (IL‐1β) and Interleukin‐18 (IL‐18), result in further upregulation of pro‐fibrotic factors, including transforming growth factor‐β (TGF‐β). This, in turn, activates myofibroblasts, accelerating ECM deposition and fibrosis formation (Artlett 2022).

The intensity of the inflammatory response is of crucial importance in the classification of different ILD subtypes. The inflammatory response in IPF patients is more severe than in cryptogenic organizing pneumonia, potentially representing a key factor underlying the differences in prognosis and corticosteroid response between the two conditions (Lu et al. 2023). Systematic anti‐inflammatory regimens are developed based on the specific type and severity of ILD, aiming to rapidly control acute inflammation and subsequently maintain long‐term immunosuppression (Yamano et al. 2018). The objective of this strategy is to swiftly manage the acute inflammatory phase prior to the initiation of long‐term immunosuppression. In the context of persistent chronic inflammation, the inflammatory resolution mechanisms within lung tissue are dysregulated, thereby preventing timely alleviation of the inflammatory microenvironment. This results in the sustained stimulation of interstitial cells, thus promoting abnormal repair responses and the progression of fibrosis. It is evident that this phenomenon ultimately results in structural damage to the lungs and a progressive decline in pulmonary function (Robb et al. 2016). Consequently, the inflammatory response not only initiates the pathogenesis of ILD, but also serves as a major driver, propelling its progression towards fibrosis. Given the central role of the inflammatory response in ILD, daily dietary intake of functional food ingredients with anti‐inflammatory properties may represent a promising adjunctive strategy for alleviating chronic pulmonary inflammation and slowing fibrosis progression.

#### 2.2.2 The Role of Oxidative Stress in ILD

Oxidative stress has been demonstrated to play a pivotal role in the genesis of numerous diseases and is intrinsically linked to the physiological processes of aging (Luo et al. 2020). As a condition highly correlated with advancing age, IPF exhibits accelerated aging due to oxidative stress. Given the persistent exposure of the lungs to hyperoxic environments, their antioxidant defense mechanisms are of particular importance. Current antioxidant therapeutic strategies for IPF are no longer limited to the administration of exogenous antioxidants, but increasingly target multiple pathways, including modulating specific oxidase activity, boosting endogenous antioxidant defenses, clearing senescent cells, and restoring mitophagy function (Otoupalova et al. 2020).

In conditions of oxidative stress, excessive production of reactive oxygen species (ROS) directly damages cell membrane structures, functional proteins, and genetic material, causing widespread oxidative damage that ultimately leads to cellular dysfunction or even apoptosis (Calleja et al. 2021). This process not only perpetuates cellular injury and inflammatory responses, but also constitutes a key factor driving the vicious cycle of oxidative stress due to the underlying imbalance in antioxidant defense systems. In patients with ILD, the antioxidant system is frequently depleted. It is recommended that future research explore more specific antioxidants that target the sources of reactive ROS production or their signaling pathways (Bast et al. 2010). In a seminal study, Tang C et al. discovered that in pemphigus‐associated ILD, anti‐Dsg1 antibody‐induced oxidative damage and immune complex deposition exacerbated pulmonary interstitial inflammation and fibrosis. The administration of molecular hydrogen therapy has been demonstrated to significantly mitigate the pathological alterations observed, through its antioxidant effects. This suggests a potential interplay between oxidative stress and immune mechanisms in ILD, and highlights the therapeutic potential of molecular hydrogen therapy (Tang et al. 2025). In a similar manner, mesenchymal stem cell‐derived extracellular vesicles have been shown to attenuate PF progression by modulating immune‐inflammatory and oxidative stress pathways (Gao et al. 2024). Oxidative stress exacerbates cellular energy metabolism disorders and cell death by inducing mitochondrial dysfunction, thereby creating a vicious cycle (Yu et al. 2024). The natural product avocado oil, a source of antioxidants, exhibits a combination of antioxidant, anti‐inflammatory, and antifibrotic effects in a model of PF induced by bleomycin (BLM) (Ochi et al. 2025). These findings provide valuable new insights into the potential of natural products as complementary therapies for ILD. Therefore, incorporating antioxidant‐rich functional foods into one's daily diet offers a practical strategy for combating oxidative stress and supporting lung health, thereby complementing existing health management approaches.

#### 2.2.3 The Role of Immunoregulation in ILD

Immune dysregulation has been demonstrated to play a complex and pivotal role in the pathogenesis and progression of ILD. The dysfunction of immune cells, including T cells, B cells, and macrophages, disrupts normal immune tolerance, thereby exacerbating inflammatory damage to the pulmonary interstitium and accelerating the fibrotic process (Zhan et al. 2025). Macrophages, acting as effector cells of the immune response, regulate the local microenvironment, thereby promoting fibrotic formation. Concurrently, the aberrant activation of lymphocytes, particularly T cells, disrupts the body's intrinsic immune tolerance equilibrium, inducing autoimmune reactions that further exacerbate interstitial lung injury (Mikhail and O'dwyer 2025).

ILD associated with systemic autoimmune diseases is characterized by the accelerated injury and fibrosis of lung tissue, which is caused by the production of various specific autoantibodies, including anti‐synthetase antibodies, and the activation of downstream inflammatory signaling pathway. In patients diagnosed with myositis‐associated ILD, those who test positive for anti‐MDA5 antibodies often exhibit symptoms of rapidly progressive ILD, accompanied by significantly elevated serum ferritin levels and refractory hypoxaemia. This suggests that immune‐mediated excessive inflammatory responses play a central role in disease deterioration (Mehta et al. 2022). In a study of anti‐synthetase syndrome‐associated ILD (ASS‐ILD), serum levels of pro‐inflammatory and anti‐inflammatory cytokines, such as IL‐1β and IL‐2, were found to be markedly elevated in comparison to healthy controls. This finding is indicative of widespread immune activation. It is noteworthy that an elevated T helper Type 17 (Th17)/regulatory T cell (Treg) ratio exhibited a strong correlation with the severity of pulmonary function impairment, thereby providing novel insights into potential biomarkers and therapeutic targets for ASS‐ILD (Wang, Li, Lv, et al. 2024).

Previously, IPF was considered primarily to result from fibroblast activation and abnormal epithelial repair. However, recent studies have revealed that immune dysregulation is an equally indispensable component in the pathogenesis of IPF. Indeed, IPF may be regarded as a disease driven by multiple immune cell types, characterized by macrophage polarization towards the M2 phenotype. This is characterized by the upregulation of marker molecules such as CD206, which promotes fibrosis through the release of factors including TGF‐β (Perrot et al. 2023). Furthermore, studies have demonstrated a marked accumulation of the CD71^−^ negative subpopulation within alveolar macrophages of IPF patients, with its frequency positively correlated with patient mortality. Concurrently, alterations in the proportion and functional abnormalities of regulatory T cells within peripheral and pulmonary tissues contribute to the regulation of the fibrotic process (Shenderov et al. 2021). In light of the aforementioned immunological mechanisms, the emergence of targeted therapeutic strategies that focus on macrophage polarization states, restoring the balance among Th1, Th2, and Th17 cells, and the targeted modulation of specific cytokines or immune checkpoints has become increasingly recognized as a highly promising new therapeutic direction for IPF and other fibrotic ILD. As a functional food, ginseng contains active compounds that regulate macrophage polarization and the balance of T cell subsets, thereby supporting immune homeostasis in the lungs and holding significant health‐promoting potential.

### 2.3 The Role of Key Signaling Pathways in ILD

Multiple signaling pathways form the molecular basis of the pathological processes underlying ILD. Ginseng extracts exert anti‐inflammatory, antioxidant, and immunomodulatory effects via these pathways, thereby promoting lung health.

#### 2.3.1 TGF‐β Signaling Pathway

The TGF‐β signaling pathway is a central regulatory element in the fibrotic process of ILD. This pathway has been demonstrated to drive pulmonary tissue remodeling and functional loss by promoting fibroblast activation and inducing epithelial‐mesenchymal transition (EMT). It also accelerates excessive ECM deposition, suppresses the self‐repair capacity of the alveolar epithelium, and inhibits endogenous anti‐fibrotic factors (Saito et al. 2018).

TGF‐β, a pivotal inducer of myofibroblast differentiation, has been shown to promote the transformation of pulmonary fibroblasts into α‐smooth muscle actin (α‐SMA) positive myofibroblasts. These cells then synthesize substantial amounts of collagen and fibronectin, among other ECM components, ultimately leading to pulmonary tissue stiffening and structural disruption (Coker et al. 2001). Moreover, as a primary driver of EMT, TGF‐β modulates this process by upregulating transcription repressors such as pulmonary interstitial cells Snail Family Transcriptional Repressor 1 while downregulating epithelial markers like E‐cadherin. This process instigates a mesenchymal phenotype in alveolar epithelial cells, thereby enhancing their migratory capacity and anti‐apoptotic properties, and thus directly contributing to fibrotic development (Shirakihara et al. 2007). It is notable that TGF‐β also suppresses normal alveolar epithelial cell proliferation, hindering effective epithelial regeneration following injury, and diminishes expression of endogenous antifibrotic factors such as hepatocyte growth factor and prostaglandin E2, thereby further exacerbating the fibrotic process. Consequently, the aberrant activation of this signaling pathway is regarded as a pivotal pathological basis for fibrotic progression in ILD (Garrison et al. 2013). In recent years, intervention strategies targeting this pathway have emerged as a research focus. For instance, thymosin β4 has been demonstrated to effectively inhibit the proliferation, migration, and activation of pulmonary fibroblasts in both in vitro and in vivo experiments by modulating the TGF‐β1 signaling pathway, while also attenuating the EMT process, thereby exhibiting promising anti‐fibrotic potential (Yu et al. 2025). These findings suggest that the TGF‐β signaling pathway is a key biological mechanism that influences the fibrotic process and promotes lung health.

#### 2.3.2 Nuclear Factor Kappa‐Light‐Chain‐Enhancer of Activated B Cells (NF‐κB) Signaling Pathway

The NF‐κB signaling pathway, acting as a pivotal hub regulating inflammatory and immune responses, participates in the expression of multiple inflammatory mediators and plays a crucial role in the pathogenesis of ILD (Xu et al. 2025). Within the pathological environment of ILD, factors such as autoantibodies, immune complexes, and various cytokines can lead to sustained activation of the NF‐κB pathway, thereby initiating the transcription of a series of pro‐inflammatory factors including Tumor necrosis factor‐α (TNF‐α) and IL‐1β. These factors not only promote the recruitment of inflammatory cells to the site of the pulmonary lesion, but also enhance their adhesion to vascular endothelium, ultimately leading to extravasation of inflammatory cells into the pulmonary interstitial space (Genovese et al. 2023). Beyond its role in regulating inflammation, NF‐κB also directly contributes to the development of PF. This pathway promotes the differentiation of pulmonary fibroblasts into myofibroblasts while inhibiting their apoptosis, thereby sustaining excessive ECM production and exacerbating tissue structural remodeling (Zhang et al. 2022).

NF‐κB is known to amplify the effects of pro‐fibrotic signaling pathways, such as TGF‐β, thereby creating a vicious cycle in which inflammation and fibrosis mutually reinforce each other (Li, Liang, et al. 2022). Recent studies have revealed that Bruton's tyrosine kinase (BTK), as a pivotal upstream kinase in the NF‐κB pathway, plays a crucial role in downstream signal transduction via its activation. Inhibiting the activation of the NF‐κB pathway significantly alleviates the resulting storm of pro‐inflammatory cytokines and the fibrotic process, whilst improving relevant markers of inflammation and fibrosis (Liu, Liu, et al. 2024). Furthermore, upon activation, NF‐κB acts as an upstream regulator of the NLRP3 inflammasome, promoting the expression of NLRP3 and pro‐IL‐1β. Following assembly, the NLRP3 inflammasome becomes fully activated, resulting in the cleavage of the Gasdermin D protein by Caspase‐1. This process instigates pyroptosis and facilitates the release of mature IL‐1β and IL‐18, thereby exacerbating local inflammatory responses. Concurrently, NF‐κB upregulates EMT‐associated transcription factors, leading to the loss of the epithelial cell marker E‐cadherin and the acquisition of mesenchymal cell characteristics, thereby further driving fibrosis progression. It is important to note that IL‐1β, released upon NLRP3 activation, can participate in a positive feedback loop, activating NF‐κB. This in turn establishes a vicious cycle of inflammation and fibrosis (Peng et al. 2020). Consequently, the inhibition of this pathway is regarded as a promising therapeutic approach for the management of inflammation and the prevention of fibrosis.

#### 2.3.3 Nuclear Factor Erythroid‐Related Factor 2 (Nrf‐2) Signaling Pathway

Nrf‐2, a core transcription factor in the cellular antioxidant defense system, plays a pivotal role in regulating the expression of multiple antioxidant enzymes and maintaining cellular redox homeostasis. Under physiological conditions, Nrf‐2 initiates the transcription of multiple antioxidant genes, including heme oxygenase‐1 (HO‐1). Nrf‐2 binds to antioxidant response elements, which enhances cellular resistance to oxidative damage (Zhang et al. 2021).

In ILD, dysfunction of the Nrf‐2 signaling pathway is closely associated with oxidative stress and the fibrotic process. In the absence of Nrf‐2 activity, or in cases where Nrf‐2 expression is inadequate, alveolar Type II epithelial cells exhibit an increased vulnerability to oxidative damage induced by external stimuli, such as radiation. This subsequently results in a deterioration of the pulmonary epithelium's capacity for self‐repair, thereby expediting the onset and progression of pathological processes, including radiation‐induced PF (Traver et al. 2017). Numerous animal studies and clinical observations indicate that naturally occurring Nrf‐2 activators, such as triterpenoids and other natural products, can effectively enhance the antioxidant capacity of lung tissue, mitigate inflammatory responses, and fibrotic lesions, demonstrating potential for supporting lung health (Ashrafizadeh et al. 2021). Furthermore, indepth investigations reveal a cross‐regulatory relationship between the Nrf‐2 and NF‐κB signaling pathways, both jointly participate in maintaining cellular redox balance and modulating inflammatory responses. In microenvironments characterized by oxidative stress and inflammation, the activation of Nrf‐2 has been shown to enhance antioxidant defenses and suppress NF‐κB‐mediated excessive inflammatory responses. This, in turn, has been shown to alleviate tissue damage and delay the progression of fibrosis (Saha and Rebouh 2023). Consequently, targeted activation of the Nrf‐2 pathway may be considered a crucial strategy for alleviating oxidative stress‐induced PF and preserving the structural and functional integrity of lung tissue.

#### 2.3.4 Mitogen‐Activated Protein Kinase (MAPK) Signaling Pathway

The MAPK signaling pathway is critically involved in multiple pathological processes associated with PF. These processes include regulating inflammatory responses, initiating EMT, and proliferating and activating fibroblasts. This pathway comprises a cascade of three core kinases, Extracellular signal‐regulated kinase 1/2 (ERK1/2, ERK), p38 mitogen‐activated protein kinase (p38MAPK), and c‐Jun N‐terminal kinase (JNK), each having distinct functional specializations (Fang and Richardson 2005).

Mechanistically, the transcription factor Forkhead Box O3 (FOXO3) has been demonstrated to inhibit fibroblast proliferation and the expression of ECM‐related genes, thereby indicating anti‐fibrotic potential. However, upon activation of TGF‐β signaling, the MAPK pathway is known to induce the phosphorylation of FOXO3, thereby diminishing its transcriptional activity and consequently releasing its inhibitory effects on cell cycle progression and ECM synthesis (Li, Li, Hu, et al. 2023). Therefore, targeted intervention in ERK1/2 signaling or its downstream events, alongside maintaining FOXO3 stability, represents a promising biological mechanism to explore for supporting lung health.

In contrast to ERK, p38MAPK is more responsive to cellular stress and inflammatory signals. Its activation promotes fibroblast proliferation and collagen deposition, as well as upregulates the expression of pro‐inflammatory factors such as TNF‐α and Interleukin‐6 (IL‐6), thereby exacerbating pulmonary inflammation and fibrosis (Nguyen et al. 2024). Notably, p38MAPK exerts dual regulatory functions on fibroblasts, simultaneously promoting collagen synthesis while inhibiting proliferation. This multifaceted action makes it a complex yet highly valuable node for understanding dietary modulation. Furthermore, recent studies have revealed that macrophages exhibit sustained activation of the MAPK signaling pathway under oxidative stress. This phenomenon may be attributable to the absence of inhibitory feedback from ANNEXIN, leading to persistent activation of p38MAPK signaling that cannot be effectively downregulated. Consequently, uncontrolled inflammatory responses drive the progression of pulmonary inflammation and fibrosis (Lee et al. 2024). Modulating the MAPK pathway through dietary intake of bioactive compounds, such as ginsenosides, may therefore offer a nutritional strategy to mitigate inflammation‐related lung damage.

## 3 Regulatory Mechanisms of Ginseng and Its Major Active Components on ILD

Advances in separation and identification techniques, coupled with research in molecular biology, have led to a shift in the understanding of ginseng's efficacy and mechanisms of action. This transition involves a shift from traditional empirical knowledge to a more scientific interpretation. The pharmacological diversity of ginseng is attributable to its complex chemical composition, which is primarily composed of ginsenosides, ginseng polysaccharides, polyacetylenes, phytosterols, and flavonoid compounds (Zhou et al. 2023). Ginsenosides, as the core active constituents, are characterized by pentacyclic and tetracyclic triterpenoid saponin structures (Geng et al. 2024). Based on differences in aglycone structure, these compounds are primarily classified into three categories. The protopanaxadiol (PPD) type includes Rb1, Rb2, Rc, Rd., Rg3, and Rh2, as well as their representative metabolite 20(S)‐PPD, which exhibits enhanced lipophilicity and improved cell membrane permeability, demonstrating significant activity in antitumour effects and apoptosis induction. Protopanaxatriol type, such as Re, Rf, Rg1, Rg2, and Rh1, has been demonstrated to exert significant effects in the domains of neuroprotection, antioxidant activity, and the enhancement of learning and memory functions. Oleanane‐type saponins, such as Ro, have been demonstrated to exhibit favorable anti‐inflammatory potential (Zhou et al. 2023). In addition to saponins, ginseng polysaccharides have been shown to possess a wide range of pharmacological activities, including immunomodulatory and antioxidant effects (Liu, Geng, et al. 2024). Notably, the source and processing methods have a substantial impact on the active constituents of ginseng. It is evident that wild ginseng typically contains higher concentrations of ginsenosides than cultivated ginseng, resulting in more pronounced immunomodulatory and anti‐inflammatory effects (Li, Wang, et al. 2021). Conversely, black ginseng undergoes significant changes in its ginsenoside composition through repeated steaming, exhibiting superior antioxidant activity compared to white and red ginseng (Metwaly et al. 2019).

**FIGURE 2:**
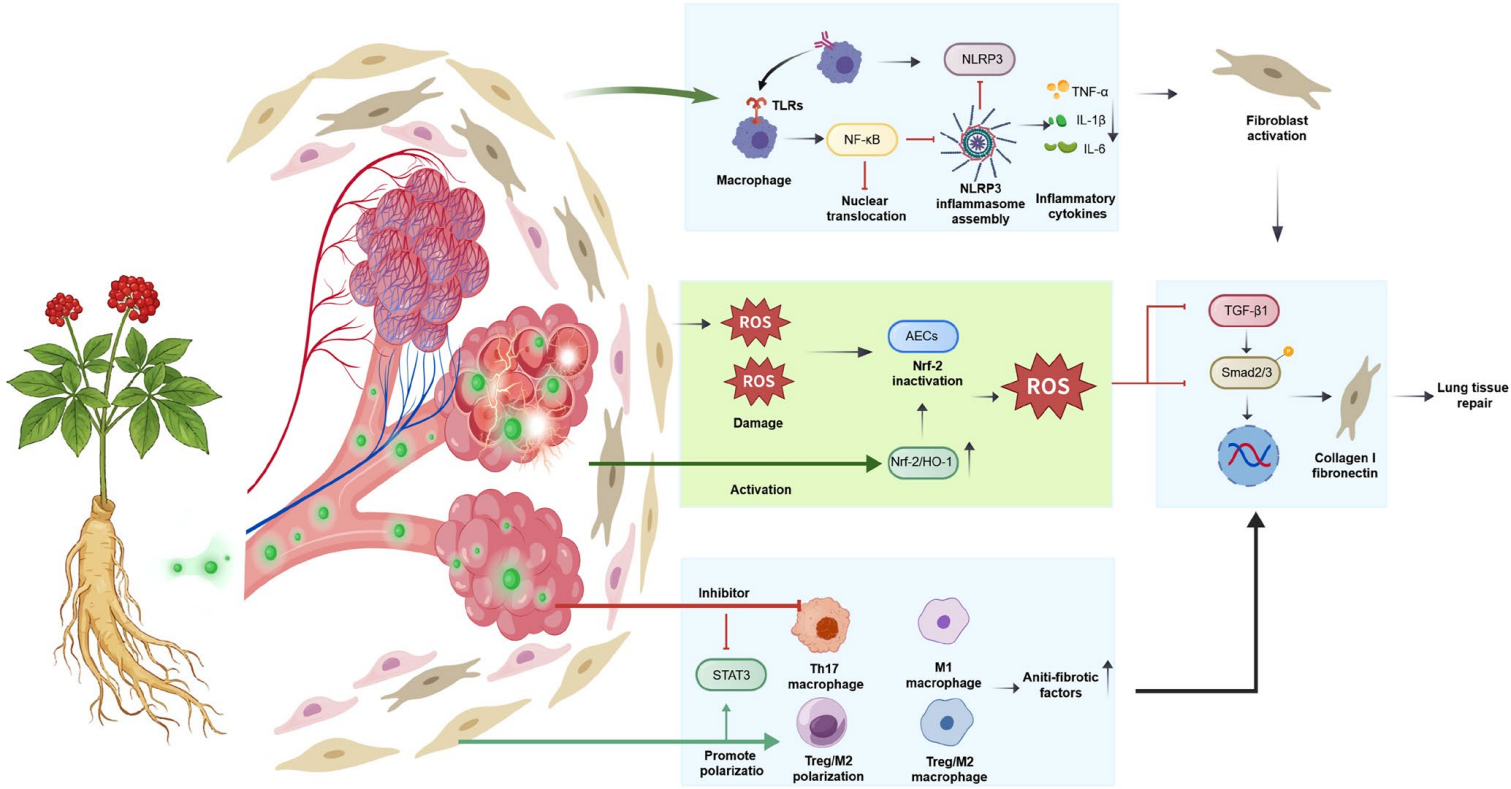
Ginseng and its active components exert anti‐inflammatory, antioxidant, and immunomodulatory effects by cross‐talking through the TGF‐β/Smad signaling pathway.

Ginseng has been shown to possess significant health‐promoting potential in fibrotic diseases across multiple organs, including the liver, lungs, myocardium, and kidneys (Liu et al. 2020). Among these, PF has been identified as the core pathological feature of ILD and the common terminal stage of various diffuse ILD. Consequently, considerable attention has been drawn to the development of prevention and treatment strategies for this condition. A plethora of studies have demonstrated that ginseng extracts have a significant impact on enhancing pulmonary tissue vitality and retarding the fibrotic process. It is worthy of note that in radiation‐induced PF models, high‐dose ginseng extracts have been demonstrated to exhibit significant antioxidant effects against radiation‐induced damage. Following pretreatment with ginseng, immunoreactivity for TGF‐β1 and the oxidative stress marker 4‐hydroxy‐2‐nonenal was markedly reduced in lung tissue, indicating its positive anti‐fibrotic effects (Jang et al. 2015). At the level of active constituents, ginseng and its bioactive components exert anti‐fibrotic actions in ILD through diverse mechanisms, operating via multi‐targeted, multi‐pathway synergistic mechanisms. Primary mechanism of action of ginseng and its active components in ILD intervention (Figure 2 and Jiang et al. (2025)).

It is important to note that most experimental data presented in this section are derived from preclinical studies using pharmacological doses, which are generally higher than typical dietary intake levels. The translation of these findings to human dietary supplementation requires caution and further investigation.

### 3.1 Anti‐Inflammatory Activity: Regulating the Pulmonary Inflammatory Microenvironment

The role of ginseng in modulating inflammatory responses has been extensively investigated, and it is recognized for its significant inhibition of inflammatory mediators such as TNF‐α and IL‐6. These factors play a crucial role in the onset and progression of chronic inflammation and autoimmune diseases (Kim et al. 2025). The anti‐inflammatory properties of ginsenosides primarily arise from their regulation of the principal inflammatory signaling pathways, such as NF‐κB and MAPK. Research indicates that multiple ginsenoside components, including Rb1 and Compound K, can inhibit NF‐κB nuclear translocation and activation by suppressing upstream events such as the phosphorylation of Interleukin‐1 Receptor‐Associated Kinase 1. Concurrently, they modulate the phosphorylation of p38 MAPK and JNK, ultimately reducing the expression of pro‐inflammatory cytokines such as TNF‐α and IL‐1β, as well as inflammatory mediators such as inducible nitric oxide synthase (iNOS) and cyclooxygenase‐2. This process thereby exhibits significant anti‐inflammatory and tissue‐protective effects (Kim et al. 2017). It is noteworthy that recent studies have elucidated a novel mechanism by which ginsenosides exert anti‐inflammatory effects by promoting inflammation resolution. M2 macrophages, defined by elevated phagocytic activity, reparative factor synthesis capacity, and pro‐inflammatory factor suppression, emerge as pivotal cells in the resolution phase. Ginsenosides have been shown to induce macrophage polarization towards the M2 phenotype, thereby actively promoting inflammation resolution. This mechanism is a complementary addition to the traditional pathway of suppressing M1 macrophage inflammatory responses. The dual regulatory mechanism of ginseng, which involves the inhibition of M1 pro‐inflammatory activity and the promotion of M2 resolution, provides a more comprehensive understanding of its anti‐inflammatory effects (Im 2020).

The function of ginseng in reducing pulmonary tissue damage is closely associated with its effective regulation of inflammatory responses. In the course of the progression of ILD, an overactivated inflammatory response is a key driver of pulmonary tissue injury and fibrosis (Selman et al. 2001). Research indicates that the active constituents in ginseng, particularly ginsenosides, can suppress the release of pro‐inflammatory cytokines through multiple pathways, thereby alleviating pulmonary inflammatory states (Ding et al. 2023). Taking total ginsenosides as an example, they exhibit multi‐targeted intervention properties in BLM‐induced PF models. This process is characterized by the simultaneous downregulation of the pro‐fibrotic factor TGF‐β1 and its downstream signaling molecules Smad2/3, concurrent with the upregulation of the inhibitory Smad7, thus effectively blocking the classical TGF‐β1/Smad signaling pathway. Furthermore, it has been demonstrated to enhance ECM metabolic equilibrium by means of coordinated reduction in the expression of matrix metalloproteinases (MMPs), in conjunction with their inhibitor tissue inhibitor of metalloproteinase‐1 (TIMP‐1). The combined effects of these dual mechanisms ultimately significantly attenuate fibrotic pathological alterations in lung tissue (Yang et al. 2019). It has been demonstrated that ginsenoside metabolite 20 (S)‐PPD exerts a direct effect on AMPK, consequently exerting a negative regulatory influence on the expression of STING, a pivotal innate immune protein. This effectively inhibits the activation of the TGF‐β1/Smad2 signaling pathway and reverses EMT, reducing pulmonary inflammation and collagen deposition, and ultimately alleviating PF (Ren, Lv, et al. 2023).

With regard to the particular saponin components, Rg1 has been shown to significantly alleviate alveolar inflammatory responses, reduce inflammatory cell infiltration, and inhibit the expression of the pro‐fibrotic factor TGF‐β1. Concurrently, it upregulates the negative regulatory protein Caveolin‐1, thereby inhibiting the transformation of fibroblasts into myofibroblasts, reducing the production of α‐SMA and hydroxyproline, and consequently suppressing collagen deposition and ECM remodeling, thus delaying the progression of PF (Zhan et al. 2016). Ginsenoside Rg3 has been demonstrated to significantly reduce TGF‐β2 and IL‐1β‐induced Smad2/3 phosphorylation, thereby blocking key endothelial‐to‐mesenchymal transition (EndMT) signaling pathways and exerting anti‐fibrotic effects (Yun et al. 2023). Furthermore, Rg3 counteracts inflammatory EndMT by upregulating miR‐139‐5p to inhibit the Akt/NF‐κB inflammatory signaling pathway. This offers a novel strategy targeting inflammatory EndMT for PF, particularly that associated with infection or chronic inflammation (Lee et al. 2020). It has been demonstrated that Ginsenoside Rg6 exerts a beneficial effect on recovery in mice suffering from lipopolysaccharide (LPS)‐induced lung injury, by reducing neutrophil infiltration and TNF‐α expression in lung tissue. Furthermore, the injection solution has been demonstrated to suppress systemic inflammatory responses by inducing the production of anti‐inflammatory factors, including IL‐10 and miR‐146a (Wang, Han, et al. 2023). It has been demonstrated that ginsenoside Rb1 significantly reduces the expression of pro‐inflammatory cytokines such as TNF‐α and IL‐6, which are induced by cigarette smoke extract. This effect is achieved by inhibiting activation of the NF‐κB signaling pathway by suppressing IκB phosphorylation and NF‐κB nuclear translocation (Li, Li, Lv, et al. 2023). Rb1 exerts its anti‐fibrotic effects primarily by specifically targeting the NLRP3/IL‐1β axis within macrophages and inhibiting its upstream NF‐κB pathway. This results in the disruption of key intercellular communications driving PF (Liu et al. 2022). Ginseng extract has been shown to inhibit the TLR4/NF‐κB pathways through a number of mechanisms. These include a reduction in the expression of Toll‐like receptor 4 (TLR4) protein, suppression of IκBα degradation, prevention of NF‐κB nuclear translocation, and reduction in the release of pro‐inflammatory factors, including IL‐1β and IL‐6. This sequence of actions has been demonstrated to block P2X7r/NLRP3 inflammasome activation, inhibit NLRP3 activation, and reduce the secretion of mature IL‐1β and IL‐18. Concurrently, it modulates the interactions between macrophages and alveolar epithelial cells, reduces ECM deposition, restores TIMP‐1/MMP‐13 balance, and promotes collagen degradation. This has been demonstrated to enhance the alveolar microenvironment, thereby alleviating cigarette smoke‐induced inflammation and PF (Dou et al. 2025). Rh2 promotes the M1 polarization of macrophages via the TLR4/PI3K/Akt/mTOR and Raf‐1/MEK/ERK pathways, thereby enhancing the inflammatory clearance capacity (Wang, Zhang, et al. 2023). Ginsenoside Re has been shown to significantly inhibit the phosphorylation of p38MAPK and JNK, as well as the nuclear translocation of NF‐κB and cellular Fos (c‐Fos) in lung tissue, thereby reducing inflammatory cell infiltration and tissue damage (Lee et al. 2018). These multi‐level synergistic regulatory effects constitute an important scientific basis for ginseng as an anti‐inflammatory functional food. The detailed experimental evidence supporting the anti‐inflammatory effects of ginseng and its components is summarized in Table 1.

**TABLE 1:** Research on the anti‐inflammatory effects of ginseng.

Ingredient	Dose	Exposure condition	Models	Results	References
Total Ginsenoside	40–160 mg/kg/day (in vivo)	Intragastric administration from the second day after model establishment, continuously for 28 days	BALB/c mice (BLM‐induced PF model)	Regulates TGF‐β1/Smad pathway; Reduces MMP‐2, MMP‐9, TIMP‐1 expression; Improves ECM metabolism imbalance.	(Yang et al. 2019)
20(S)‐PPD	2 μM (in vitro); 10–40 mg/kg/day (in vivo)	In vitro: co‐incubation with TGF‐β1 for 48 h; In vivo: intragastric administration 7 days after BLM induction, continuously for 14 days	Mouse alveolar epithelial cells (MLE‐12); C57BL/6 mice (BLM‐induced PF model)	Activates AMPK, downregulates STING; Inhibits TGF‐β1/Smad2 pathway; Reverses EMT; Reduces lung inflammation and collagen deposition.	(Ren, Lv, et al. 2023)
Ginsenoside Rg1	18–72 mg/kg/day (in vivo)	Oral administration the next day after BLM induction, continuously for 7–28 days	Sprague–Dawley rats (BLM‐induced PF model)	Downregulates TGF‐β1, upregulates Caveolin‐1; Inhibits fibroblast‐to‐myofibroblast transformation; Reduces α‐SMA and hydroxyproline levels; Suppresses collagen deposition and ECM remodeling.	(Zhan et al. 2016)
Ginsenoside Rg3	10 μg/mL (in vitro); 5 mg/kg/day (in vivo)	In vitro: 24 h pre‐treatment before TGF‐β2/IL‐1β co‐stimulation; In vivo: intragastric administration from the day of BLM induction, continuously for 27 days	Human pulmonary arterial endothelial cells; C57BL/6N mice (BLM‐induced PF model)	Inhibits Smad2/3 phosphorylation; Blocks EndMT; Reduces collagen deposition and alleviates PF.	(Yun et al. 2023)
Ginsenoside Rg3	10 μg/mL (in vitro)	Co‐treatment with iE‐DAP for 2–6 days	Human umbilical vein endothelial cells	Upregulates miR‐139‐5p; Inhibits Akt/NF‐κB inflammatory pathway; Blocks NOD1‐mediated inflammatory EndMT; Preserves endothelial phenotype	(Lee et al. 2020)
Ginsenoside Rb1	10 μM (in vitro); 20 mg/kg/day (in vivo)	In vitro: 6 h pre‐treatment before CSE exposure; In vivo: intraperitoneal injection from day 15 to day 28 after COPD model establishment	Human bronchial epithelial cells (BEAS‐2B); Sprague–Dawley rats (cigarette smoke + LPS‐induced COPD model)	Inhibits IκB phosphorylation and NF‐κB nuclear translocation; Blocks NF‐κB pathway activation; Reduces CSE‐induced TNF‐α, IL‐6, IL‐1β expression	(Li, Li, Lv, et al. 2023)
Ginsenoside Rb1	20 μM (in vitro); 20 mg/kg/day (in vivo)	In vitro: 24 h pre‐treatment before LPS/ATP stimulation; In vivo: 24 h after BLM induction, daily administration until euthanasia	THP‐1‐derived macrophages, MRC‐5 fibroblasts; C57BL/6 mice (BLM‐induced PF model)	Targets macrophage NLRP3/IL‐1β axis; Inhibits upstream NF‐κB pathway; Blocks macrophage‐fibroblast crosstalk; Reduces α‐SMA and collagen I levels; Alleviates pulmonary inflammation and fibrosis	(Liu et al. 2022)
Ginseng Aqueous Extract	300 mg/kg/day (in vivo)	Daily gavage before CS exposure, continuous treatment for 11 weeks	BALB/c mice (cigarette smoke‐induced PF model)	Inhibits TLR4/NF‐κB pathway (reduces TLR4, IRAK4 expression, inhibits IκBα degradation and NF‐κB nuclear translocation); Blocks P2X7r/NLRP3 inflammasome activation (downregulates NLRP3, ASC, P2X7r); Reduces IL‐1β, IL‐6, IL‐18 secretion; Restores TIMP‐1/MMP13 balance, reduces α‐SMA and collagen deposition; Regulates macrophage‐alveolar epithelial cell crosstalk to improve alveolar microenvironment	(Dou et al. 2025)
Ginsenoside Rh2	10–20 μM (in vitro); 25–100 mg/kg/day (in vivo)	In vitro: co‐incubation with LPS/ATP or macrophage‐conditioned medium; In vivo: continuous administration during model intervention	RAW264.7 macrophages, A549 cells, BALB/c mice (OVA‐induced asthma model)	Activates TLR4/PI3K/Akt/mTOR and Raf‐1/MEK/ERK pathways; Promotes macrophage M1 polarization (upregulates CD16/32, downregulates CD206); Enhances inflammation clearance; Reduces VEGF, MMP‐2/9 levels	(Wang, Zhang, et al. 2023)
Ginsenoside Re	6–50 mg/kg/day (in vivo) 50 ~ 100 μM (in vitro)	In vivo: 1 h before intranasal LPS administration, oral gavage; In vitro: co‐incubated with IL‐1β/LPS for 4 to 24 h	ICR mice (LPS‐induced acute lung injury model); A549 cells, MH‐S alveolar macrophages	Inhibits p38MAPK, JNK phosphorylation and NF‐κB, c‐Fos nuclear translocation; Reduces inflammatory cell infiltration and alveolar wall hyperplasia; Attenuates lung tissue injury; inhibits IL‐6 and NO production	(Lee et al. 2018)
neutrophil membrane‐engineered *Panax ginseng* root‐derived exosomes	20 μg/kg (in vivo); 50 μg/mL (in vitro)	In vivo: tail vein injection for 3 consecutive days starting at 24 h after LPS (10 mg/kg) intraperitoneal injection; In vitro: co‐incubation with LPS (50 μM) for 24 h after 24 h pre‐treatment with exosomes	C57BL/6J mice (LPS‐induced sepsis‐associated acute lung injury model); MLE‐12	Downregulates Drp‐1 phosphorylation and NLRP3 inflammasome activation reduces ASC, Caspase‐1, IL‐1β levels. Reduces lung inflammation, lowers TNF‐α, IL‐1β, IL‐6	(Ma et al. 2024)

### 3.2 Antioxidant Activity: Enhancing Pulmonary Endogenous Defense Mechanisms

The antioxidant properties of ginseng are primarily attributed to its bioactive constituents, most notably ginsenosides and polysaccharides. A substantial body of research has demonstrated that ginseng can effectively reduce oxidative stress‐induced cellular damage. This process involves the direct scavenging of free radicals, the inhibition of hydrogen peroxide production, and enhancing the activity of endogenous antioxidant enzymes, including superoxide dismutase (SOD) and glutathione peroxidase (GPx) (Soyuçok et al. 2024). In a range of experimental models, ginseng has shown beneficial effects on various oxidative stress biomarkers. The findings of this study demonstrate that the administration of this compound leads to a substantial reduction in serum malondialdehyde (MDA) levels, while concurrently enhancing total antioxidant capacity and upregulating the expression of SOD, glutathione, and glutathione reductase. It is noteworthy that when the intervention period extends beyond 4 weeks, ginseng significantly augments the activity levels of GPx and catalase (Ren, Lin, et al. 2023).

In models of respiratory disease, red ginseng has been shown to alleviate oxidative stress by inhibiting the production of ROS, while also suppressing NF‐κB phosphorylation and iNOS expression. This, in turn, has been demonstrated to improve asthma symptoms through dual antioxidant and anti‐inflammatory pathways (Kim, Kim, Kim, et al. 2022). Ginsenoside Rg1 has been found to enhance the antioxidant and anti‐apoptotic capacity of Alveolar Epithelial Cell Type II (AECII) by activating the Nrf‐2/HO‐1 signaling pathway, while promoting their differentiation into AECI. The ultimate objective is to facilitate the acceleration of the repair and regeneration of lung tissue (Kobayashi et al. 2020). Ginsenoside Rb1 has been shown to promote Nrf‐2 nuclear translocation, thus activating downstream antioxidant genes such as HO‐1, whilst concomitantly reducing MDA levels, a lipid peroxidation product, and increasing SOD and catalase activity. The net effect of these processes is to effectively alleviate oxidative stress induced by cigarette smoke extract (Li, Li, Lv, et al. 2023). Furthermore, panaxydol, a compound isolated from ginseng roots, has been shown to activate the Keap1‐Nrf‐2/HO‐1 pathway, leading to the upregulation of HO‐1. In addition, panaxydol has been observed to restore glutathione levels and GPX4 activity. This process has been shown to inhibit the accumulation of MDA, a lipid peroxidation product, and ferroptosis. This subsequently reduced levels of key pro‐inflammatory cytokines such as TNF‐α and IL‐1β in bronchoalveolar lavage fluid and cell supernatants, thereby alleviating pulmonary inflammation (Li, Lu, et al. 2021). The investigation revealed that the engineered exosome, derived from the root of *Panax ginseng* , when modified using neutrophil membranes, exhibited enhanced targeting of lung tissue. The delivered microRNAs have been shown to specifically suppress NADPH oxidase 4 expression, thereby downregulating Dynamin‐related protein 1 phosphorylation and NLRP3 inflammasome activation, ultimately improving mitochondrial function and alleviating oxidative stress (Ma et al. 2024). The administration of black ginseng extract resulted in a mitigated endothelial barrier disruption, as evidenced by the scavenging of PM2.5‐induced ROS and the inhibition of p38MAPK activation. Concurrently, it activates the PI3K/Akt signaling pathway, thereby enhancing endothelial cell survival and barrier integrity. This has been shown to significantly reduce pulmonary vascular leakage, leukocyte infiltration, and inflammatory cytokine release, thereby exerting protective and reparative effects on lung tissue (Lee et al. 2019). These findings suggest that, as a source of dietary antioxidants, ginseng helps strengthen pulmonary defense against external oxidative challenges. The specific experimental data on the antioxidant effects of ginseng and its components are presented in Table 2.

**TABLE 2:** Research on the antioxidant effects of ginseng.

Ingredient	Dose	Exposure condition	Models	Results	References
Korean Red Ginseng	100–300 mg/kg/day (in vivo)	From days 18 to 23 of model establishment	Female BALB/c mice (OVA‐induced allergic asthma model)	Inhibits ROS production to alleviate oxidative stress; Reduces NF‐κB phosphorylation and iNOS expression; Decreases Th2 cytokines (IL‐4, IL‐5, IL‐13) and IgE levels; Attenuates airway hyperresponsiveness and mucus overproduction	Kim, Kim, Kim, et al. (2022)
Ginsenoside Rg1	18–72 mg/kg/day (in vivo); 10–20 μM (in vitro)	In vitro: pre‐incubation with AECII cells for 24 h before oxidative stress induction; In vivo: oral administration post BLM injury for 7–28 days	Sprague–Dawley rats (BLM‐induced fibrosis model); mouse AECII cell line	Activates Nrf‐2/HO‐1 signaling; Enhances AECII antioxidant and anti‐apoptosis ability; Promotes AECII differentiation into AEC I; Accelerates lung tissue repair and regeneration	Kobayashi et al. (2020)
Ginsenoside Rb1	10 μM (in vitro); 20 mg/kg/day (in vivo)	In vitro: 6 h pre‐treatment before CSE exposure; In vivo: intraperitoneal injection from day 15 to day 28 after COPD model establishment	BEAS‐2B; Sprague–Dawley rats (cigarette smoke + LPS‐induced COPD model)	Promotes Nrf‐2 nuclear translocation; Activates downstream antioxidant genes (HO‐1, NQO‐1, Gclc); Reduces MDA content; Improves SOD and CAT activities; Alleviates CSE‐induced oxidative stress	Li, Li, Lv, et al. (2023)
Panaxydol	20 mg/kg (in vivo); 10–40 μg/mL (in vitro)	In vivo: intraperitoneal injection from day 0 to day 2 before LPS tracheal injection (10 μg/mouse); In vitro: co‐treatment with LPS (10 μg/mL) for 24 h (pre‐treatment with SnPP/ML385 for pathway inhibition)	C57BL/6 mice (LPS‐induced acute lung injury model); BEAS‐2B	Activates Keap1‐Nrf‐2/HO‐1 pathway; Upregulates HO‐1, restores GSH level and GPX4 activity; Inhibits lipid peroxidation and ferroptosis; Lowers BALF/cell supernatant TNF‐α, IL‐1β, IL‐6 levels; Ameliorates lung pathological damage, edema and inflammatory cell infiltration	Li, Lu, et al. (2021)
neutrophil membrane‐engineered *Panax ginseng* root‐derived exosomes	20 μg/kg (in vivo); 50 μg/mL (in vitro)	In vivo: tail vein injection for 3 consecutive days starting at 24 h after LPS (10 mg/kg) intraperitoneal injection; In vitro: co‐incubation with LPS (50 μM) for 24 h after 24 h pre‐treatment with exosomes	C57BL/6J mice (LPS‐induced sepsis‐associated acute lung injury model); MLE‐12	Delivers miRNA‐182‐5p to specifically inhibit NOX4 expression; Improves mitochondrial function; Alleviates oxidative stress (reduces MDA/ROS/MPO, increases SOD/GSH‐Px), ameliorates lung wet/dry ratio and pathological damage	Ma et al. (2024)
Black Ginseng Extract (BGE)	0.05–0.6 g/kg/day (in vivo); 0.2 mg/mL (in vitro)	In vivo: peroral administration for 10 days before 10 consecutive days of intratracheal PM_2.5_ (1 mg/kg) challenge; In vitro: 6 h pre‐treatment before 6 h PM_2.5_ (1 mg/mL) exposure	BALB/c mice (PM_2.5_‐induced pulmonary injury model); mouse lung microvascular endothelial cells	BGE protects the integrity of the pulmonary vascular barrier by scavenging reactive oxygen species, inhibiting the p38MAPK pathway, and activating the Akt pathway, thereby mitigating PM_2.5_‐induced pulmonary inflammation and injury	Lee et al. (2019)

### 3.3 Immunomodulatory Activity: Promoting Immune Homeostasis

As a functional food, ginseng exerts bidirectional immunomodulatory effects aimed at restoring homeostasis rather than simply enhancing immune activity. Ginseng‐derived miR‐156, when administered orally, is absorbed and modulates immune‐ and metabolism‐related gene expression in mice through cross‐regulatory mechanisms. In relation to immune regulation, miR‐156 has been demonstrated to directly promote proliferation and phagocytic function in RAW264.7 macrophages, while concomitantly increasing TNF‐α secretion. These findings suggest the potential for immunostimulatory effects (Wang, Li, Ruan, et al. 2024). Furthermore, ginseng and its fermentation products can be converted by gut microbiota into the rare saponin Compound K, which exhibits higher bioavailability. Compound K modulates the composition of the intestinal flora by promoting the growth of beneficial bacteria and inhibiting pathogenic bacteria. Additionally, ginseng and its fermentation products can enhance mucosal and systemic immune responses by regulating immune cells, such as macrophages and T cells, and key signaling pathways, including NF‐κB and MAPK (Ye et al. 2023).

It has been postulated that ginseng and its active constituents may also modulate the functions of various immune cells. In macrophages, ginsenoside Rg3 has been shown to enhance Fcγ receptor‐mediated phagocytic activity by activating the ERK1/2 and p38MAPK pathways (Xin et al. 2019). In dendritic cells, ginseng polysaccharides promote maturation through the upregulation of surface markers such as CD86, accompanied by the secretion of IL‐12 and TNF‐α (Lee et al. 2009). In murine models, supplementation with the ginseng extract CVT‐E002 has been shown to consistently elevate natural killer (NK) cell counts, suggesting a potential for CVT‐E002 to function as an innate immune enhancer by augmenting NK cell activity (Miller et al. 2012). Regarding adaptive immunity, ginsenosides modulate T‐cell function by influencing IL‐2 production through the NF‐κB pathway, thereby participating in Th1/Th2 balance regulation. Concurrently, their constituents promote B‐cell proliferation and antibody class switching, increasing antigen‐specific IgA production. Collectively, these mechanisms serve to reinforce the multifaceted regulatory effects of ginseng on the immune system (Ratan et al. 2021). With regard to the antimicrobial effects of ginseng and its active constituents, it has been demonstrated that these substances disrupt cell membrane structures, thereby leading to dissipation of membrane potential and a reduction in membrane fluidity. Concurrently, they inhibit quorum sensing and biofilm formation while modulating host immune responses, thereby exhibiting significant inhibitory effects against both viruses and bacteria (Sung and Lee 2008). Such effects position ginseng as a potential immunomodulator, demonstrating particular promise in addressing immunodeficiency and chronic diseases. In the context of autoimmune diseases, the focus extends beyond enhancing immune function to restoring systemic balance. Specifically, ginseng extracts have been shown to correct the Th17/Treg cell imbalance by inhibiting phosphorylation in the STAT3 signaling pathway, thereby suppressing pathogenic Th17 cells and promoting regulatory Treg cells (Jhun et al. 2014). A substantial body of clinical research has indicated that the long‐term ingestion of ginseng can result in a reduced incidence of certain cancers, including gastric and lung cancer. This phenomenon may be attributable to ginseng's ability to induce apoptosis in tumor cells, impede angiogenesis, and modulate the tumor microenvironment (Yun et al. 2001). Ginseng has been shown to possess antiviral, antibacterial and antimicrobial properties, and thus may be employed as an immunomodulator in the prevention and treatment of bacterial, viral and autoimmune diseases (Song et al. 2026).

Ginseng and its bioactive constituents act in the lungs via multiple mechanisms, which encompass the enhancement of both innate and adaptive immunity, the suppression of pathogens, and the modulation of inflammatory pathways. Alveolar macrophages have been identified as being central to the early inflammatory response in ILD, and ginsenosides have been shown to regulate innate immune responses while inhibiting abnormal macrophage activation. Ginsenoside Rg1 exerts bidirectional immunomodulatory effects on macrophages by suppressing inflammatory responses via NF‐κB inhibition and promoting protein translation through PI3K/Akt/mTOR activation. It suppresses LPS‐induced transcription of TNF‐α and IL‐6 via NF‐κB inhibition while also enhancing TNF‐α protein translation through PI3K/Akt/mTOR activation (Wang et al. 2014). Ginsenoside Rg3 has been demonstrated to induce M2 polarization in macrophages, accelerating inflammatory resolution while simultaneously inhibiting M1 macrophage polarization and reducing pro‐inflammatory mediator production (Kang et al. 2018). A common ultimate outcome of immune dysregulation is fibrosis. Ginsenosides have been shown to exert a direct effect on effector cells, thereby inhibiting fibroblast activation and EMT. Red ginseng extract has been shown to enhance the survival rate of A549 cells following Influenza A virus subtype H1N1 (H1N1) infection by boosting protective immunity through increased T‐cell and CD11c^+^ cell activity. It also promotes Interferon‐γ production and reduces inflammatory cell infiltration (Lee et al. 2014). The combination of ginseng and vitamin C has been demonstrated to enhance the activation of immune cells, including NK cells and T cells. In addition, this combination has been shown to inhibit the progression of the lytic cycle of various viruses, including H1N1, and to alleviate pulmonary inflammation (Kim et al. 2016). Research findings demonstrate that ginsenoside Re enhances vaccine immunogenicity by simultaneously boosting both Th1 and Th2 immune responses. This results in increased antibody titres and improved cellular and humoral immune responses (Song et al. 2010). Ginseng and its primary active constituents have been demonstrated to exert systemic regulatory effects, ranging from innate immune macrophage polarization to the rebalancing of adaptive immune T‐cell subsets. This encompasses the inhibition of key upstream inflammatory pathways and the blockade of downstream fibroblast activation and EMT processes. This systemic immunomodulatory effect confers unique value to ginseng in supporting pulmonary immune health. The experimental evidence for the immunomodulatory effects of ginseng and its components is systematically summarized in Table 3.

**TABLE 3:** Research on the immunomodulatory effects of ginseng.

Ingredient	Dose	Exposure condition	Models	Results	References
Red Ginseng Extract	100–500 μg/mL (in vitro); 25 mg/kg/day (in vivo)	In vitro: 24 h pre‐incubation before H1N1 infection, continuous treatment for 48 h post‐infection; In vivo: oral administration for 30 consecutive days before intranasal infection with 1.0 LD_50_ H1N1	A549 cells; 9–10 months old female BALB/c mice (H1N1 model)	In vitro: Improves A549 cells viability post‐H1N1 infection, reduces virus‐induced CPE, inhibits influenza‐induced IL‐6/IL‐8 expression and intracellular ROS generation; In vivo: Enhances IFN‐γ production in lung/BAL fluid (upregulates CD8^+^ T cell and CD11c^+^ cell IFN‐γ secretion), reduces bronchiolar inflammatory cell infiltration and intrabronchiolar exudate area, exerts immunomodulatory	Lee et al. (2014)
Red Ginseng	In vitro: VC 50–100 μM + RG 250–2500 μg/mL; In vivo: VC 0.035–0.35 mg/day + RG 250 μg/day (for 3 weeks)	In vitro: 24 h pre‐treatment before TPA stimulation (BCBL‐1) or 48 h co‐incubation (PBMC/NK cells); In vivo: 3‐week supplementation before intranasal H1N1 (10,000 PFU) infection	Human PBMCs/NK cells, KSHV‐infected BCBL‐1 cells; Gulo^−^/^−^ mice (vitamin C‐deficient, H1N1 infection model)	In vitro: Synergistically upregulates CD25/CD69 on CD3^+^ T cells and NK cells, increases NKp46 expression; suppresses KSHV lytic genes (RTA, K8, ORF45) and reduces BCBL‐1 viability In vivo: Enhances splenic NK cell NKp46 expression and plasma IFN‐γ production, reduces H1N1 lung viral PFU, alleviates lung inflammation/edema, improves survival rate and reduces body weight loss	Kim et al. (2016)
Ginsenoside Re	25–100 μg/dose (in vivo, combined with 10 ng HA antigen)	Subcutaneous immunization twice at 3‐week intervals (Weeks 0 and 3), sampling at 2 weeks post‐boost	Female ICR mice (split inactivated H3N2 influenza vaccine immunization model)	Enhances humoral immunity: increases serum HA‐specific IgG titer, elevates IgG1/IgG2a/IgG2b/IgG3 isotypes, boosts HI titer Activates cellular immunity: promotes splenocyte proliferation to Con A/LPS, upregulates splenocyte secretion of IFN‐γ (Th1) and IL‐5 (Th2), induces balanced Th1/Th2 immune responses	Song et al. (2010)

## 4 Research on the Use of Ginseng in ILD

### 4.1 Association Between Ginseng Intake and Lung Health

As a medicinal and edible functional food, ginseng has been increasingly incorporated into dietary supplements and routine health management in recent years. A substantial body of research has demonstrated that ginseng and its primary constituents possess both anti‐inflammatory and anti‐fibrotic properties, indicating considerable potential as a functional food approach for ILD‐related health outcomes. For instance, ginsenosides and metabolites such as 20(S)‐PPD have been shown to reduce ECM deposition by inhibiting the TGF‐β1/Smad pathway, thereby delaying airway remodeling (Ren, Lv, et al. 2023). Ginsenoside Rg1, by modulating signaling pathways such as p38MAPK/NF‐κB, has been shown to reduce goblet cell proliferation and mucus secretion. It has been demonstrated that Rg2, Rg3, Rh1, Rb1, and certain compound extracts are capable of balancing Th1/Th2 immune responses and inhibiting multiple inflammatory cytokines, including IL‐4 and IL‐6. This process of suppression of airway inflammation is a highly effective mechanism that has been observed in a number of studies (Guo et al. 2025). These core pharmacological actions provide a scientific foundation for investigating ginseng's clinical efficacy in alleviating cough and sputum production symptoms, as well as reducing the frequency of acute exacerbations. Further studies indicate that red ginseng extracts alleviate pulmonary inflammatory responses by inhibiting the TGF‐β1/Smad pathway, demonstrating potential for improving PF and protecting lung function. This offers a strategy for mitigating respiratory damage caused by environmental pollutants such as PM2.5 (Kim et al. 2023). Furthermore, metabolomics and 16S rRNA sequencing studies have shown that the ginsenoside metabolite PPD alleviates pulmonary fibrosis in preclinical models by modulating gut microbiota and sphingolipid metabolism, and by targeting SPHK1 and G6PD in lung tissue. This reveals a ‘gut‐lung axis’ mechanism underlying the action of ginseng, offering a new perspective for combining PPD with existing anti‐fibrotic therapies (Ruan et al. 2024). These studies lay the groundwork for the use of ginseng as a functional food in individuals with ILD; regular dietary intake helps maintain overall lung health, suggesting its potential as a supportive dietary component in managing disease progression.

### 4.2 The Combined Use of Ginseng as a Dietary Supplement With Other Treatments

Ginseng is often combined with other health approaches to enhance its benefits and reduce potential adverse effects. When combined with platycodon, ginseng significantly reduces inflammation and PF induced by cigarette smoke, by inhibiting the TLR4–P2X7r/NLRP3 signaling pathway. This results in a reduction of ECM deposition and inflammatory responses, thus exerting a synergistic effect in enhancing therapeutic outcomes (Dou et al. 2025). The combination of ginseng and Inula japonica has been shown to significantly inhibit the fibrotic process in pulmonary fibroblasts. This inhibition is achieved by suppressing the TGF‐β1/Smad pathway, thereby mitigating the toxicity observed with *Inula helenium* monotherapy (Jung et al. 2024). The ginseng‐rich traditional Chinese medicine compound Jin Shui Huan Xian Formula has been the subject of experimental validation, which has demonstrated its efficacy in reducing oxidative stress by upregulating Nrf‐2 signaling. This, in turn, has been shown to inhibit fibroblast differentiation towards myofibroblasts (Bai et al. 2018). The Maimendong Decoction, a medicinal concoction comprising ginseng components, has been shown to alleviate PF by inhibiting the PI3K/Akt/FOXO3a signaling pathway, modulating M2 macrophage polarization, and reducing the release of pro‐fibrotic factors (Shuangshuang et al. 2024). The findings, when considered collectively, provide substantiated evidence for the therapeutic potential of ginseng in the context of ILD. In combination with pharmaceutical agents such as pirfenidone and prednisone, ginseng has been observed to augment their anti‐inflammatory effects, enhance pulmonary function in patients with IPF, and improve quality of life (Lao et al. 2024). As anti‐fibrotic agents that have been approved for use, pirfenidone and nintedanib have been observed to induce certain tolerances and adverse reactions. It is evident that ginseng possesses immunomodulatory and anti‐inflammatory properties, which have been demonstrated to mitigate the occurrence of adverse effects associated with pharmaceutical interventions, while concomitantly enhancing patient tolerance (Xia et al. 2023). These findings provide a rationale for the use of ginseng as a dietary supplement.

### 4.3 Safety and Tolerability of Ginseng as a Dietary Supplement

Despite the numerous pharmacological properties of ginseng as a traditional Chinese medicine, its potential application in ILD has attracted increasing attention in recent years. When taken as a dietary supplement within the recommended intake range, ginseng is safe and well tolerated. Nevertheless, given its complex composition, there remain concerns about the safety of long‐term use and potential drug interactions (Fan et al. 2024). Side effects are usually mild and include nausea, diarrhea, and allergic reactions (Chen et al. 2021). Although some studies have observed reversible hepatotoxicity following intravenous administration of high‐dose ginsenosides, these effects occur at exposure levels far exceeding those achieved with standard oral doses and are therefore not relevant to typical dietary intake. Nevertheless, long‐term or high‐dose use of ginseng extract supplements, particularly in individuals with pre‐existing liver disease, warrants appropriate health monitoring (Gao et al. 2011). Another study demonstrated that the primary active component of ginseng, 20(S)‐ginsenoside Rg3, induced side effects when administered via long‐term, high‐dose intramuscular injections. These side effects were largely confined to reversible local irritation and dose‐dependent immune stimulation, including leukocytosis, neutrophilia, increased spleen weight, and mild renal weight changes. All observed alterations were fully reversible after treatment cessation, indicating a favorable safety profile with low toxicity risk. However, the study also highlighted the critical role of the administration route and dosage (Liu et al. 2012). Consequently, when evaluating the safety of ginseng and its constituents, factors such as specific saponin types, administration methods, dosage, and treatment duration should be carefully considered. Furthermore, attention to standardized extraction methods and quality control indicators has the potential to enhance both the efficacy and safety of ginseng preparations (Li, Jiang, et al. 2022). Although no universally accepted daily intake standard exists, most clinical studies and regulatory guidelines suggest that a daily intake of 0.5–3 g of dried ginseng root powder or its equivalent is safe for long‐term use as a dietary supplement (Li, Wang, Xu, et al. 2023). In summary, dietary consumption of ginseng is safe for the general population. However, potential safety concerns should be considered for specific groups, including pregnant women, individuals taking anticoagulant medications, and those using high‐concentration supplements for prolonged periods or at excessive doses.

## 5 Conclusion

ILD is a significant public health concern, underscoring the need for safe and effective health promotion strategies. As a traditional medicinal and edible plant, ginseng is a functional food rich in bioactive compounds. By modulating multiple signaling pathways—including TGF‐β/Smad, NF‐κB, Nrf2/HO‐1, and MAPK—these compounds exert anti‐inflammatory, antioxidant, and immunomodulatory effects, thereby helping to maintain lung health.

It is worth noting that most current evidence stems from preclinical studies, while population‐based studies—particularly rigorously designed nutritional intervention studies—remain limited. Future research should focus on well‐designed, nutrition‐oriented human studies to clarify the health effects of ginseng across different populations. Standardization of the dosage and formulation of ginseng extracts and supplements is essential, as is distinguishing between dietary intake and pharmacological intervention. Furthermore, the value of ginseng as an adjunct to health management in specific ILD subtypes warrants exploration. Through these efforts, this ancient functional food can be more effectively integrated into modern health promotion practices.

## Author Contributions

**Jiahui Li:** writing – original draft. **Guixin Di:** data curation. **Yanxin Li:** visualization. **Ziyuan Wang:** resources. **Haijiao Wang:** data curation. **Keju Wang:** investigation. **Mengzhu Chen:** formal analysis. **Tan Wang:** supervision, methodology, writing – review and editing.

## Funding

This work was supported by the National Traditional Chinese Medicine and Western Medicine Clinical Collaboration Project for Major and Intractable Diseases (Grant No. ZDYN‐2024‐A‐022), the Project of Construction of National Famous Traditional Chinese Medicine Inheritance Workshop (National Chinese Medicine Office Human Education Letter [2022] No. 245) and the 2026 Doctoral Research Innovation Capability Improvement Project of Changchun University of Chinese Medicine (2026KYCX04).

## Conflicts of Interest

The authors declare no conflicts of interest.

## Data Availability

The authors have nothing to report.
